# Genome-Wide Identification and Role of the *bHLH* Gene Family in *Dendrocalamus latiflorus* Flowering Regulation

**DOI:** 10.3390/ijms251910837

**Published:** 2024-10-09

**Authors:** Mei-Yin Zeng, Peng-Kai Zhu, Yu Tang, Yu-Han Lin, Tian-You He, Jun-Dong Rong, Yu-Shan Zheng, Ling-Yan Chen

**Affiliations:** 1 College of Landscape Architecture and Art, Fujian Agriculture and Forestry University, Fuzhou 350002, China; 2 College of Forestry, Fujian Agriculture and Forestry University, Fuzhou 350002, China

**Keywords:** *bHLH* transcription factor, period of flowering, bamboo flower development, *Dendrocalamus latiflorus*

## Abstract

The basic helix–loop–helix (*bHLH*) gene family is a crucial regulator in plants, orchestrating various developmental processes, particularly flower formation, and mediating responses to hormonal signals. The molecular mechanism of bamboo flowering regulation remains unresolved, limiting bamboo breeding efforts. In this study, we identified 309 *bHLH* genes and divided them into 23 subfamilies. Structural analysis revealed that proteins in specific *DlbHLH* subfamilies are highly conserved. Collinearity analysis indicates that the amplification of the *DlbHLH* gene family primarily occurs through segmental duplications. The structural diversity of these duplicated genes may account for their functional variability. Many *DlbHLHs* are expressed during flower development, indicating the *bHLH* gene’s significant role in this process. In the promoter region of *DlbHLHs*, different homeopathic elements involved in light response and hormone response co-exist, indicating that *DlbHLHs* are related to the regulation of the flower development of *D. latiflorus*.

## 1. Introduction

Bamboo species exhibit synchronized flowering and die-off cycles, controlled by complex genetic mechanisms [1]. The collective death of bamboo has resulted in massive losses for forest institutions and private cultivators and has created a serious ecological crisis [2,3,4,5,6]. The molecular regulation of bamboo flowering is a critical research challenge.

Molecular studies have identified numerous flowering-related genes involved in pathways like photoperiod, vernalization, circadian rhythms, temperature, aging, hormones, and sugar production [7,8]. The *bHLH* domain comprises approximately 60 amino acids, characterized by two amphipathic α-helices that are separated by a variable-length loop region [9]. Members of the *bHLH* family collaborate with various other proteins to facilitate the regulation of flower development in plants [10]. For example, *MdbHLH4* in apples promotes flowering by promoting GA accumulation and FLOWERING LOCUS T (*FT*) gene expression [11]. A new *bHLH* transcription factor in chrysanthemum, *CmbHLH110*, can speed up flowering [12]. Blue light receptors called cryptochromes (*CRY1/2*) prevent hypocotyl elongation and regulate the onset of flowers [13], CRYPTOCHROME-INTERACTING BASIC HELIX–LOOP–HELIX 1 (*CIB1*) is a bHLH protein that interacts with CRY2 to promote *FT* transcription. In response to blue light, A protein complex that can be formed by *CRY2*, *CIB1*, and CONSTANS (*CO*) can then encourage floral initiation [14,15]. In brief, the transcription factor gene family *bHLH* is involved in controlling the growth and development of plants, abiotic stress, and metabolic network responses [16]. However, the regulatory interactions between bHLH transcription factors (TFs) and hormones in bamboo remain unclear.

*D. latiflorus* is a clumpy, woody bamboo that blooms sporadically [17]. It has great economic value and is commonly grown in tropical and subtropical regions [18,19]. *D. latiflorus* often blooms sporadically, and the seed-setting rate of the plant flowering in its natural state is very low [20], which seriously restricts the genetic improvement process of *D. latiflorus* [21,22]. To understand the significance of the *bHLH* gene family in regulating the flower development of *D. latiflorus*, the *bHLH* gene family was identified and analyzed in this study. Subsequently, the potential molecular function of *bHLHs* was inferred through differentially expressed genes at four different stages of flower development. This study provides a foundation for understanding the molecular mechanisms of flower development in bamboo.

## 2. Results

### 2.1. bHLH TFs in D. latiflorus

We discovered 309 members of the *bHLH* gene family in *D. latiflorus* (Table S1), designated as *DlbHLH1*-*DlbHLH309* under their respective chromosomal locations. Moreover, a comprehensive physicochemical characterization was performed on the 309 DlbHLH proteins. The encoded proteins by the aforementioned DlbHLH proteins exhibited a size range of 88 (*DlbHLH72*) to 1628 (*DlbHLH195*) amino acids, with molecular weights spanning from 9820.08 Da to 183,630.63 Da and isoelectric points (pI) ranging from 4.61 to 11.05. All proteins were unstable in vitro, according to the instability index, except for *DlbHLH27*, *DlbHLH36*, *DlbHLH57*, *DlbHLH129*, and *DlbHLH139*. Subcellular localization predictions showed that 260 *DlbHLHs* were nuclear, while smaller numbers were found in the chloroplasts (24), cytosol (9), endoplasmic reticulum (5), peroxisome (4), mitochondria (4), plasma membrane (2), and vacuole membrane (1). The prediction results of the transmembrane structure (TM) showed that none of the other family members had transmembrane structures except *DlbHLH88* and *DlbHLH189*, which had one and two transmembrane structures, respectively. None of the *DlbHLH* members had signal peptide sequences according to signal peptide prediction analytics. The diversity of subcellular localization indicates that members of the *DlbHLH* gene family conduct a wide range of biological functions.

### 2.2. Phylogenetic Analysis of the DlbHLHs

**Figure 1:**
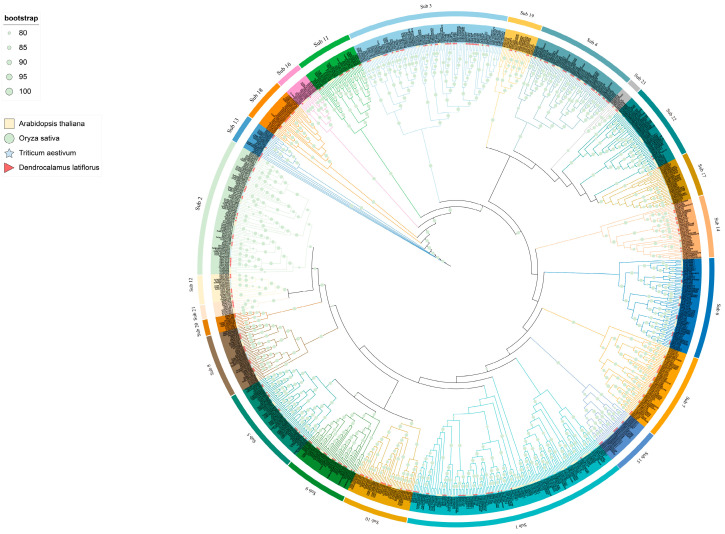
Maximum likelihood (ML) phylogenetic tree of bHLH proteins from *D. latiflorus*, *A. thaliana*, *O. sativa*, and *T. aestivum*. Sub is short for subfamily.

To elucidate the phylogenetic relationship among the *DlbHLHs*, *bHLHs* from *D. latiflorus* (309), *Arabidopsis thaliana* (158) [23], *Triticum aestivum* (324) [24], and *Oryza sativa* (190) [25] were used to construct an ML tree (Figure 1). The phylogenetic tree containing 981 *bHLH* genes was divided into 23 subfamilies based on the taxonomy and topology suggested in earlier works [26,27] (Table S1). These subfamilies ranged in size from 2 to 37 members, with subfamily 3 having the greatest number of *DlbHLHs* (37), and subfamilies 16, 17, and 22 having just 1 *DlbHLH*. In multiple clusters during evolution, The *bHLH* gene family demonstrated substantial species bias, indicating that particular gene duplication happened after species divergence [28].

### 2.3. Gene Structure and Conserved Motifs of DlbHLHs

**Figure 2:**
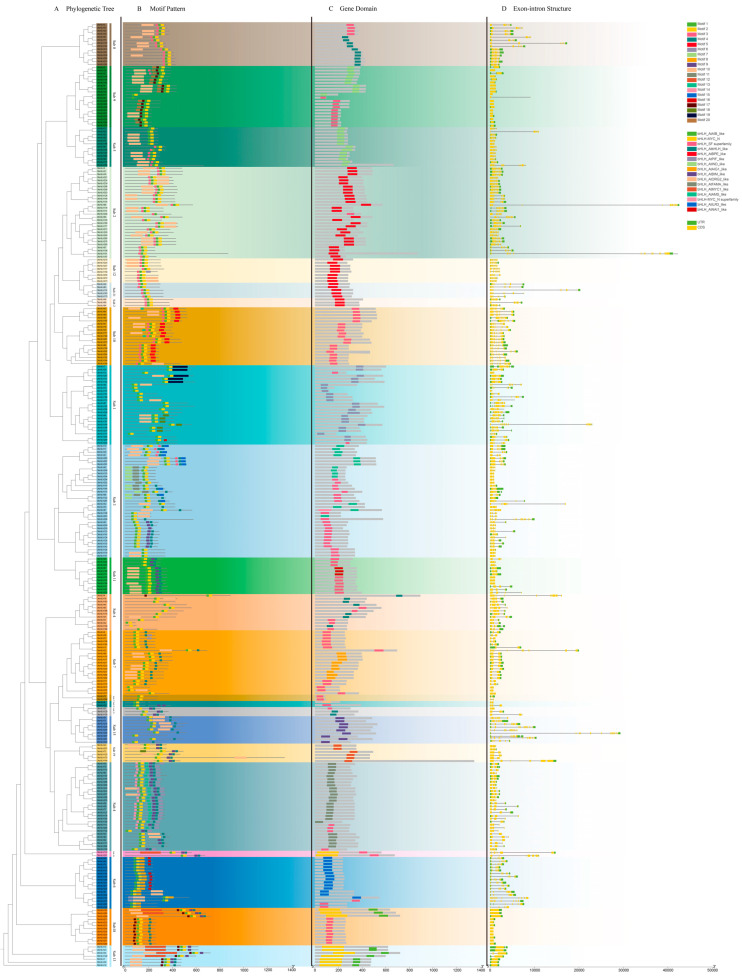
The phylogenetic relationship, conserved motifs, and gene structure of *DlbHLHs*. (**A**): The maximum likelihood (ML) phylogenetic tree of DlbHLH protein was constructed using the full-length sequence of 1000 bootstrap repeats; (**B**): Distribution of conserved motifs of DlbHLH protein. A total of 20 patterns were predicted, and the scale bar represented 200 aa. (**C**): bHLH domain distribution of *DlbHLHs*; (**D**): Genetic structure of *DlbHLHs*, including coding sequences (yellow rectangle) and untranslated regions (UTRs, green rectangle). The scale bar represents 10,000 bp.

The gene structure and conserved sequences of the 309 *DlbHLH* genes based on their evolutionary relationships were analyzed and provided (Figure 2). Using the MEME, a conserved pattern search on 309 *DlbHLH* genes identified 20 conserved motifs (motifs 1–20; Figure 2B). *DlbHLH* genes from the same subfamily shared similar types, amounts, and distributions of conserved motifs. Motifs 1, 2, and 10 are found in the majority of *DlbHLH* genes, while others are found only in a few. For example, (motifs 1, 2, 3, 10) and (motifs 1, 2, 4, 9, 10) are present in every member of the families of subfamilies 8 and 11, respectively. Afterward, we evaluated the DlbHLH protein domains and exhibited the locations of the bHLH-conserved domains that were matched to the *D. latiflorus* gene structure (Figure 2C). The findings showed there were 16 distinct bHLH domain types with considerable diversity, and that, with few exceptions, gene members belonging to the same subfamily had similar *bHLH* domain types.

We also examined the architecture of the *DlbHLH* genes, which ranged in length from 472 bp (*DlbHLH157*) to 42,590 bp (*DlbHLH88*) (Figure 2). The number of exons in the 309 *DlbHLHs* ranged from 1 to 17 (Figure 2D). The amount of *DlbHLHs* with three CDSs (51) is the highest, followed by *DlbHLHs* with seven CDSs. *DlbHLH191* has the greatest number of exons and introns among all members, with 17 and 16, respectively. *DlbHLH178* had as many as five UTRs, indicating that its alternatively spliced version may be the most complicated [29].

### 2.4. Chromosomal Location, Collinearity, and Evolution Analysis of DlbHLHs

Gene duplication is an important first step in the process of extending the functioning of a multigene family [30]. In each subgenome, we found that the *DlbHLHs* are evenly distributed on eleven or twelve chromosomes. Nevertheless, each chromosome has an unequal distribution of *DbHLH* numbers. chr2.1 and chr25.1, which belong to the B and C subgenomes, respectively, had the largest number of *DlbHLHs*, with sixteen each. The second most abundant are chr1.1 and 7.1, each with fifteen *DlbHLHs*. ch35.1 has the lowest number of *DlbHLHs*, only two.

**Figure 3:**
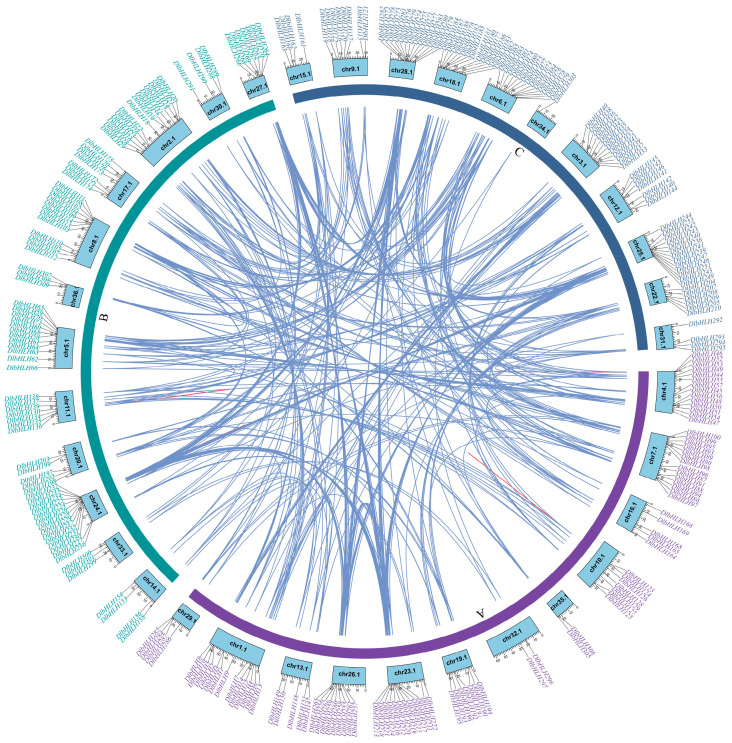
Chromosomal location and collinearity analysis of *DlbHLH* family genes. The letters A–C represent the subgenomic distribution of *DlbHLHs*. Blue-purple for subgenome A, green for subgenome B, and blue for subgenome C. Chromosomes are represented by light blue boxes. Segmental duplication genes are connected with blue lines. Tandem duplication genes are connected with red lines.

To investigate the *DlbHLH* family gene duplication events, homology analysis identified 379 fragment repeat pairs and 4 tandem repeat pairs (Figure 3; Table S2). Subfamily 4 has the largest number of duplicate gene pairs (48 pairs, all of which are fragment copies). The results indicate that *DlbHLH* fragment replication events are the primary factors influencing the evolution of *DlbHLHs*. Through a comparison of mutation rates between Ka (nonsynonymous) and Ks (synonymous), the selection pressure of gene duplications was assessed [31], so we computed the Ka/Ks ratio for all 383 gene pairs (Table S3). All gene pairs had Ka/Ks ratios below 1, with most under 0.5, suggesting that *DlbHLH* gene pairs underwent purifying selection during evolution.

**Figure 4:**
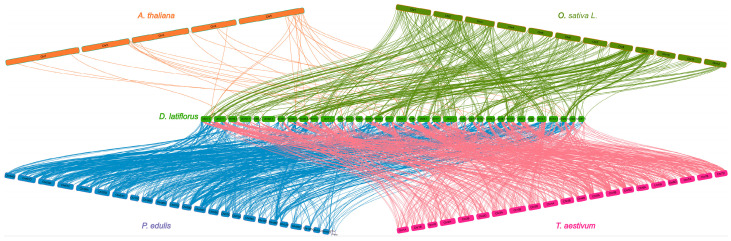
*bHLH* gene collinearity analysis of *D. latiflorus* and 4 representative plants.

We chose four sample species for collinearity comparative study to investigate the gene replication timing of *DlbHLHs* and determine its phylogenetic relationship: *Arabidopsis thaliana*, *Triticum aestivum*, *Oryza sativa*, and *Phyloglostachys edulis* (Figure 4). Collinear analysis of different species is a way to study their evolution and affinities [32]. A total of 877 *PebHLHs* were collinearly correlated with the bamboo genome, followed by wheat (832), rice (399), and *Arabidopsis* (32) (Table S4). Among them, There are 23 *DlbHLHs* (*DlbHLH4*, *DlbHLH7*, *DlbHLH24*, *DlbHLH26*, *DlbHLH47*, *DlbHLH54*, *DlbHLH59*, *DlbHLH70*, *DlbHLH116*, *DlbHLH137*, *DlbHLH151*, *DlbHLH169*, *DlbHLH199*, *DlbHLH208*, *DlbHLH218*, *DlbHLH220*, *DlbHLH226*, *DlbHLH237*, *DlbHLH245*, *DlbHLH247*, *DlbHLH284*, *DlbHLH285*, and *DlbHLH289*) (Table S4-2) in all four representative species. The presence of collinear associations between species suggests that genes in these *bHLH* gene families are essential in evolution.

### 2.5. Cis-Acting Elements in DlbHLH Promoters

**Figure 5:**
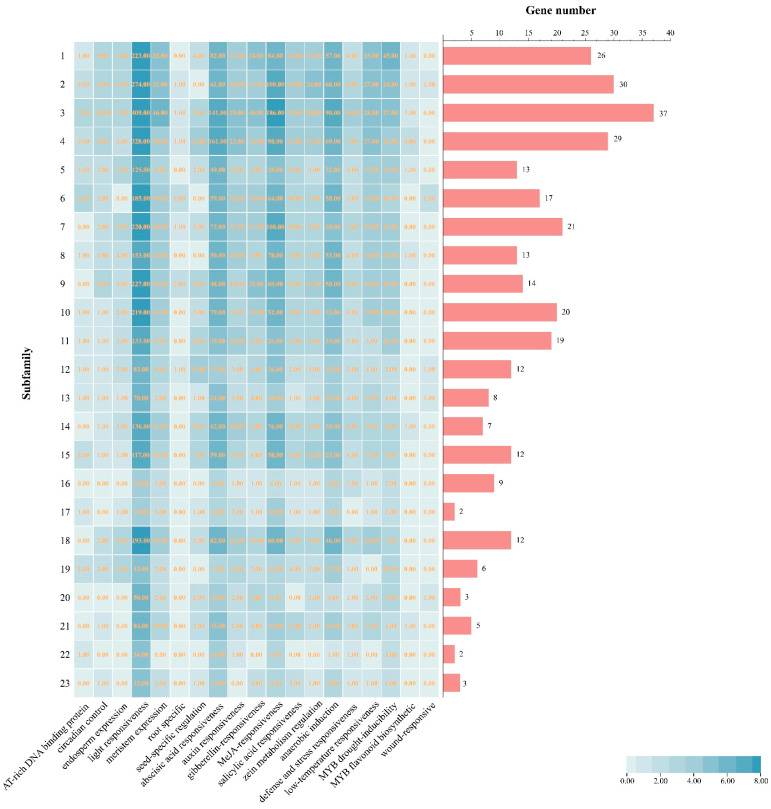
The number of CREs on putative promoters of *DlbHLHs*.

Cis-regulatory elements (CREs) contribute to development, and one common reason for evolutionary change is their divergence [33]. A total of 8488 CREs were predicted in the promoter regions of *DlbHLHs* (Table S5), with 19 representative CREs shown in Figure S1. Among these, *DlbHLH238* had the most *CREs*, a total of 62 elements. The overall number of CREs was categorized into three major groups (Figure S1), and the number of CREs differed within subfamilies (Figure 5). Growth and development (3909) comprised the following categories: root-specific (9, 0.2%), circadian control (55, 1.4%), seed-specific regulation element (87, 2.22%), endosperm expression element (65, 1.66%), light-responsive element (3375, 86.33%), meristem expression element (282, 7.21%), and AT-rich DNA binding protein (36, 0.9%). According to Figure 5, within the promoter area of every *DlbHLH*, CREs linked to light responsiveness (such as the GATA-motif, GATA-motif, G-box, ACE, and Box I, etc.) are extensively expressed. Apart from components that react to light, there are additional regulatory components necessary for growth and development. They might be missing in many subfamilies such as AT-rich for AT-rich DNA binding protein, GCN4_motif for endosperm, RY-element for seed, and circadian for circadian control.

The second class was related to hormone responsiveness (3166), including abscisic acid-responsive (1175, 37.11%), auxin-responsive (222, 7%), gibberellin-responsive (244, 7.70%), MeJA-responsive (1206, 38.01%), salicylic acid-responsive (151, 4.77%), and zein metabolism regulation (168, 5.30%). MeJA-responsive elements (CGTCA-motif and TGACG-motif) and abscisic acid-responsive elements (ABREs) are widely present in the promoter regions of almost all *DlbHLH* subfamilies. CREs associated with salicylic acid response are present in most subfamilies except subfamilies 20 and 22. CREs associated with the auxin reaction (AuxRE, AuxRR-core, TGA-element, and TGA-box) are found in most subfamilies except subfamily 23 in their promoter region. Gibberellin-responsive elements (GARE-motif, TATC-box, and P-box) are present in the promoter region of 163 of the 309 *DLbHLHs* except for the subfamily 22. In addition, many *DlbHLHs*, such as *DlbHLH8*, *DlbHLH12*, *DlbHLH15*, *DlbHLH42*, *DlbHLH54*, *DlbHLH65*, *DlbHLH78*, *DlbHLH86*, *DlbHLH143*, *DlbHLH216*, *DlbHLH233*, and *DlbHLH289*, in the promoter regions contain multiple identical hormone responses, which suggests that the specific hormones may have a more rapid and stronger reaction. At the same time, some *DlbHLH* response elements, such as *DlbHLH12*, *DlbHLH102*, *DlbHLH109*, *DlbHLH143*, *DlbHLH152*, *DlbHLH297*, *DlbHLH219*, and *DlbHLH238*, in the promoter regions contain a variety of hormones, implying that they might be involved in some hormone regulatory networks.

The third class was stress-responsive CREs (1413), containing anaerobic induction (775, 54.85%), defense and stress-responsive elements (98, 6.94%), low-temperature responsive elements (246, 17.40%), MYB drought inducibility (277, 19.60%), MYB flavonoid biosynthetic elements (11, 0.78%), and wound-responsive elements (6, 0.42%). Among these, CREs for anaerobic induction and MYB drought inducibility are present and abundant in all subfamilies. CREs associated with defense and stress-responsive elements are present in most subfamilies except subfamily 17. CREs associated with defense and low-temperature responsive elements are present in most subfamilies except subfamilies 19 and 22. Furthermore, several subfamilies are shown to have higher amounts of stress-related CREs.

Previous studies have shown that JASMONATE ASSOCIATED MYC2-LIKE1 (JAM2) negatively regulates JA response by antagonizing MYC2 [34]. We identified some homologous genes of *JAM2*, namely *DlbHLH1*, *DlbHLH14*, *DlbHLH19*, *DlbHLH38*, *DlbHLH132 DlbHLH148*, *DlbHLH183*, and *DlbHLH285*, and these gene promoter regions have Me-JA response elements. The findings showed that there was substantial variation in the number and makeup of cis-regulatory elements in the promoter regions of various *DlbHLHs*, both within and between subfamilies. This discovery implies that a wide range of CREs associated with hormones, plant growth, developmental processes, and stress response control the expression of *bHLH* genes in *D*. *latiflorus*.

### 2.6. Transcription Factor Binding Sites (TFBSs) in DlbHLH Promoters

The binding sites for 42 TF families were identified in the promoters of the *DlbHLH* family members (Figure S2). The greatest number of C2H2 binding sites was observed to be 289. This was followed by MYB and NAC, with 235 and 228, respectively, while the lowest number was recorded for YABBY, with a mere 3. In addition, TFBSs in each *DlbHLH* promoter differ in type, number, and location. The promoter region of *DlbHLH98* has 24 binding sites, while *DlbHLH244* has only 4 binding sites, namely C2H2, MYB, B3, and ERF.

### 2.7. Protein–Protein Interaction Network of the DlbHLH Members

Based on the homologous alignment of *DlbHLH* in *Arabidopsis*, we constructed a protein–protein interaction network between *DlbHLHs* (Figure S3). The results showed that there was an interaction between the DlbHLH proteins. *AT4G0050* (*PIF8*) and *AT2G14760* (*bHLH94*) exhibit a direct interaction. In addition, there are some polygenic interactions. Examples include *AT3G47640* (*bHLH47*), *AT2G22759* (*bHLH18*), *AT5G5960* (*bHLH41*), and *AT1G68810* (*bHLH30*). Notably, we identified several genes involved in hormone-regulated flower development pathways. For example, *AT1G32640* (*MYC2*) has been reported to be involved in jasmonic acid-mediated *Arabidopsis* flowering inhibition [35]. *AT5G08130* (*BIM1*) is a Brassinosteroid signal transduction element that synergistically regulates pollen fertility with SQUAMOSA PROMOTER BINDING PROTEIN-LIKE 8 (*SPL8*) [36]. *AT1G01260* (*JAM2*) regulates petal senescence by JA [37]. These genes are directly or indirectly related to each other.

### 2.8. Expression Patterns of DlbHLHs in Four Flower Developmental Stages

**Figure 6:**
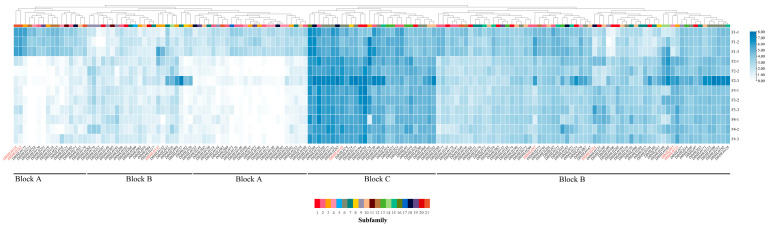
Hierarchical clustering of *DlbHLH* expression at different flowering stages. The heatmap was drawn based on the log2 (TPM+1) values. TPM: transcripts per million mapped reads. The size and color scales represent expression levels from low to high. The eight genes highlighted in red are selected for qRT-PCR.

**Figure 7:**
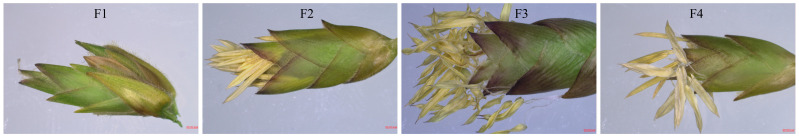
*D. latiflorus* flower can be divided into four stages: young bud stage (**F1**), flowering mid-term stage (**F2**), full-bloom stage (**F3**), and fading stage (**F4**).

The expression profiles of 309 *DlbHLHs* in four phases of flower development were described using RNA-seq data to investigate the potential functions of the *DlbHLHs* (Figure 6 and Figure 7). After excluding genes with low levels of expression, 156 *DlbHLHs* remained. Using hierarchical clustering, the *DlbHLHs* could be divided into four blocks (block A–C); genes from the same subfamilies may have diverse expression patterns. The *DlbHLHs* in different blocks exhibited disparate patterns of expression in space and time: (A) *DlbHLHs* in block A are preferentially expressed in bud stage, including genes from subfamily 2, subfamily 3, subfamily 4, subfamily 5, subfamily 7, subfamily 9, subfamily 10, subfamily 11, subfamily 12, subfamily 18, subfamily 19, and subfamily 21. (B) Most of the *DlbHLHs* in region B were expressed in four flower developmental stages, among which *DlbHLH135* was highly expressed in bud stage and *DlbHLH143* was highly expressed in middle-flower development, exhibiting a trend in initially growing and then reducing during bloom development (C). The majority of *DlbHLHs* in the C region show high expression levels at all four phases of floral development. This finding suggests that these genes may play a function in floral formation. More importantly, CRY2-INTERACTING BHLH 4 (*CIB4*) has been shown to participate in FT transcription and thus to promote flowering [38]. Many *DlbHLHs* have been identified as *CIB4* homologs, such as *DlbHLH21*, *DlbHLH39*, *DlbHLH97*, *DlbHLH118*, and so on. Among them, *DlbHLH97* and *DlbHLH118* are only significantly expressed in the bud stage, showing a developmental stage-specific expression pattern. We identified *BIM1* as a pollen sterility gene among homologous genes [36], respectively, *DlbHLH51*, *DlbHLH71*, *DlbHLH223*, *DlbHLH228*, *DlbHLH228*, *DlbHLH238*, and *DlbHLH259*. In addition to *DlbHLH238* in block C, which is highly expressed in all four stages of flower development, other homologous genes are located in block B. *FBH1* is a *bHLH*-related protein and a transcriptional activator of the *CO* gene. It can favorably control CO-mediated blooming time [39]. *DlbHLH214* of block C was highly expressed at all four stages of flower development, indicating similar functions in *D. latiflorus*. *MYC2* regulates the flowering time of *Arabidopsis* by inhibiting *FT* transcription in the JA pathway [35,40]. In Figure 6, four homologous *DlbHLHs* of *MYC2* (*DlbHLH233*, *DlbHLH246*, *DlbHLH264*, and *DlbHLH271*) are expressed in different stages of flower development priority. Moreover, *DlbHLH246*, *DlbHLH264*, and *DlbHLH271* promoter regions exist in the Me-JA responsive elements. These results show a similar function in the regulation of *D. latiflorus* flower development.

### 2.9. Validation of DlbHLH Expression

**Figure 8:**
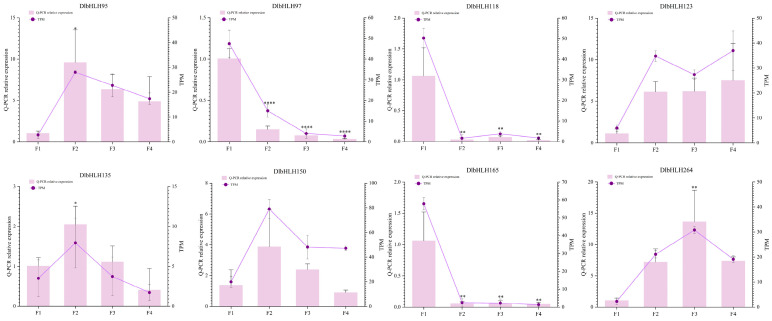
Relative expression patterns of eight selected *DlbHLH* genes in four flower developmental stages. Asterisks indicate significant differences in qRT-PCR relative expression level compared with those of the early stage of young bud stage (F1). (* *p* < 0.05, ** *p* < 0.01, **** *p* < 0.0001).

To verify the reliability of the transcriptome data, qRT-PCR was used to analyze the expression profiles of eight representative *DlbHLHs* at four flower developmental stages (Figure 8; Table S7). Consistent with the transcriptome data, *DlbHLH95*, *DlbHLH123,* and *DlbHLH264* were highly expressed in late flower development, and *DlbHLH97*, *DlbHLH118,* and *DlbHLH165* were significantly highly expressed in the F1 stage. And *DlbHLH135* and *DlbHLH150* were highly expressed in all four flower developmental stages; both of these genes showed the trend in first increasing and then decreasing, suggesting that they are involved in the four flower developmental stages.

## 3. Discussion

An increasing body of information indicates that *bHLH* TFs participate in the diverse developmental processes of eukaryotes [41,42,43,44]. *D. latiflorus* is the most widely cultivated clumping bamboo species in southern China and is of significant economic value for both human consumption and wood production [45]. The flowering period of *D. latiflorus* is long and irregular [17,22]. The establishment of its genome has paved the way for the resolution of the previously intractable issue of flowering patterns [21,46]. Although numerous *bHLH* genes in other plants have been examined, not much is known about the *bHLH* genes in *D. latiflorus*. We identified 309 *bHLH* genes in the *D. latiflorus* genome, categorizing them into 23 subfamilies according to phylogenetic relationships. The results indicated significant variation in the structure and function of bHLH TFs in different plants by comparing what is similar and different to the categories of other plant species [16]. The conserved motifs and gene structures of genes belonging to the same subfamily were shown to be similar (Figure 2). Motifs 1, 2, and 10 were conserved in almost all bHLH proteins and were critical for the operational specificity of these transcription factors [47]. Overall, the *DlbHLH* gene family is diverse and complex, with subfamily-specific structural traits supporting their phylogenetic categorization. In contrast, structural variability across distinct *DlbHLHs* may reflect the functional variety in the *bHLH* gene family.

Tandem or segmental duplication is the most commonly evaluated mechanism for gene family expansion [48]. *DlbHLH* genes were found to be extensively distributed across all subgenomes according to chromosome location. A total of 384 gene pairs (379 segmental repeats and 4 tandem repeats) with gene repetition time were discovered in *D. latiflorus*, showing that the growth of the *DlbHLH* gene family may be predominantly through fragment repeats. The Ka/Ks ratio for all gene pairings was less than one, indicating that purifying selection had a larger role than positive selection in the evolution of the *D. latiflorus bHLH* gene family. Among the plants used in the collinearity study, *Phyllostachys edulis* and *D. latiflorus* have the closest evolutionary relationship. The bulk of *DlbHLH* members are found in close clades with the *bHLHs* of rice and wheat, although their genetic lineage from *Arabidopsis* is comparatively distant. Structural research may also provide useful details on evolutionary duplication moments. Structural analysis can yield significant insights into evolutionary replication events. Certain genes that repeat have comparable patterns of expression and belong to the same subfamily. This observation indicates potential functional redundancy among them [49]. *DlbHLH21* and *DlbHLH39*, for example, share a similar motif composition, *bHLH* domain type, and gene structure, as well as similar expression patterns. As mentioned in the previous section, *DlbHLH21* and *DlbHLH39* are both homologous genes of *CIB4*, so we speculated that they may have functional redundancy in participating in *FT* transcriptional activation to promote flowering. However, many duplicate gene pairs show distinct expression patterns. For example, *DlbHLH124* and *DlbHLH63* have distinct exon–intron architectures and expression patterns. We postulate that structural alterations cause variances in the expression of these duplicated genes, leading to functional diversity.

*D. latiflorus* is significant economically, but it also possesses the unique characteristics of rapid growth and a lengthy flowering interval, pollen sterility, and so on [50,51,52]. Identifying the mechanism that controls floral growth is therefore extremely important. The *bHLH* transcription factor is essential for plant growth and development. It is involved in a variety of physiological processes and has a wide range of functions in plant hormone synthesis and metabolism [10,53]. Interestingly, we identified several *BIM1* homologs, a *bHLH* family gene that regulates pollen fertility. In the future, in situ hybridization experiments can be conducted to explore their temporal and spatial expression in anthers. In addition, we also found some homologous genes involved in hormone-regulated flowering pathways, especially jasmonic acid. Specifically, the mechanism of how the hormone interacts with the *DlbHLH* gene to regulate flowering remains to be further verified.

There are many factors regulating plant flowering, and the process is affected by light, temperature, and endogenous signal transduction. We found that light-responsive elements are present in almost all *DlbHLH* promoter regions that regulate photocontrolled transcriptional activity. A large number of *DlbHLHs* are highly expressed in some stages of flower development, which may act as a photoperiodic regulator of flowering in *D. latiflorus*. According to previous research, hormones play a crucial role in regulating bamboo flowering [54]. We found 3166 hormone-associated cis-acting elements, covering all *DlbHLHs*. This implies a potential link between *DlbHLHs* and hormonal response. In addition, abscisic acid has been observed to regulate the photoperiodic pathway in response to flowering, particularly under conditions of abiotic stress. In conditions of drought, the ABA hormone negatively regulates flowering through a pathway that requires the SOC1 protein [55], and jasmonic acid has been shown to cause pollen sterility [56]. Many *DlbHLH* promoter regions contain either abscisic acid-responsive elements (287 of 309) or jasmonic acid-responsive elements (245 of 309). This could explain why bamboo has more flowers but lower pollen and seed-setting rates. It would be beneficial for future studies to place a greater emphasis on the investigation of the regulatory mechanisms of endogenous hormones in the development of bamboo flowers. The unique flowering characteristics of bamboo may be influenced by chromosome polyploidy, and some genes related to flowering pathways have been positively selected over a long evolutionary time scale. To sum up, the *DlbHLH* genes may play a key role in the development of *D. latiflorus* flowers. However, the exact physiological mechanisms underlying their function require further experimental studies for elucidation.

## 4. Materials and Methods

### 4.1. Plant Materials

In this study, the *D. latiflorus* flowers from three flowering clumps in four development stages (F1, young bud stage; F2, flowering mid-term; F3, full-bloom stage, and F4, fading stage) were collected from a Baisha state-owned forest farm in Fuzhou, Fujian province of China (26°20′ N, 119°07′ E), in July 2023 (Figure 7); three biological replicates were collected for each sample. Before picking the flowers, alcohol was evenly sprayed on their surface to achieve thorough coverage. To ensure thorough disinfection, the alcohol was applied to the floral surfaces for 30 s to 1 min. The alcohol-sprayed flowers were then gently immersed in deionized water for approximately 2 min to remove any remaining alcohol and any dead microbes from the surface. The harvested flowers were immediately combined and flash-frozen in liquid nitrogen. They were stored at −80 °C.

### 4.2. RNA-Seq and Transcriptome Data Analysis

The RNA prep Pure Plant Kit (Tiangen, Beijing, China) was utilized to extract the total RNA from the flowers. Afterward, 2% agarose gel electrophoresis was used to evaluate the quality of the total RNA. A NanoPhotometer^®^ spectrophotometer (IMPLEN, Westlake Village, CA, USA) and a Qubit^®^ RNA assay kit with a Qubit^®^ 2.0 fluorometer (Life Technologies, Carlsbad, CA, USA) were used to measure the concentration of RNA. An Agilent^®^ Bio-analyzer 2100 system (Agilent Technologies, Santa Clara, CA, USA) was used to measure RNA integrity using an RNA Nano 6000 assay kit (Agilent Technologies, Santa Clara, CA, USA). A benchmark RNA integrity number of 7 was established as the benchmark for quality evaluation. For Illumina^®^, the NEB-Next^®^ UltraTM RNA library prep kit (NEB, Ipswich, MA, USA) was utilized during the library creation process. Using the Illumina 6000 platform, all samples underwent sequencing to provide 150 bp paired-end reads. We processed raw RNA-Seq reads using fastp v0.23.2 [50], eliminating sequences with adapters, bases with Qphred ≤ 20, and N-base percentages larger than 15%. The HiSAT2 software v2.0.1 [57] was used to map the clean RNA-seq reads to the *D. latiflorus* genome with default parameters. FeatureCounts [58] was used to quantify the mapped reads, and DESeq2 [59] (|log2 (Fold Change)| ≥ 1 and Padj ≤ 0.05) was used to screen for differentially expressed genes. The TPM value of the *DlbHLHs* was taken from all transcriptome data and normalized to log2 (TPM+1), and heat maps were created using TBtools [60].

### 4.3. qRT-PCR Validation

The same sample used for RNA-seq was utilized for qRT-PCR to confirm the expression of *DlbHLHs* in other tissues. RNA was extracted from four flower stages using the Trelief Hi-Pure Pure Plant RNA Kit (Tiangen, Beijing, China) and reverse transcribed into cDNA with the PrimeScript^™^ RT reagent kit (Perfect Real Time, Takara, Japan). Quantitative real-time PCR analysis of 8 selected *DlbHLHs* was performed using SYBR Green premix Ex Taq Kit (TaKaRa, Dalian, China) on an Applied Biosystems 7500 Real-Time System (Applied Biosystems, Foster City, CA, USA). The qRT-PCR amplification sequence was as follows: 95 °C pre-denaturation for 5 s, 95 °C denaturation for 30 s, 60 °C annealing for 30 s, 72 °C extension for 30 s, and 40 cycles. The GAPDH gene was used as an internal reference [61]. Three replicates of each gene were carried out, and the fold change was calculated using the 2^−ΔΔCT^ method. The primers were designed through the NCBI primer design online (https://www.ncbi.nlm.nih.gov/tools/primer-blast/ (accessed on 25 July 2024)). The primers used in this work are listed in Table S6.

### 4.4. Identification of D. latiflorus bHLH Transcription Factor Family

*Arabidopsis bHLH* sequences were obtained from the TAIR database (https://www.arabidopsis.org/ (accessed on 28 June 2024)), as queries used local alignment search tools (BLAST) [62] against *D. latiflorus*. The HMM profile of the bHLH transcription factor gene family (PF00010) was downloaded from Pfam (https://www.ebi.ac.uk/interpro/entry/pfam/ (accessed on 1 July 2024)) [63]. HMMER [64] search was used to search the bHLH transcription factor family in the *D. latiflorus* genome with default parameters. Consequently, the integrity of the presumed bHLH transcription factor family was further examined by InterPro [65].

### 4.5. Bioinformatic Analysis of D. latiflorus bHLH Transcription Factor Family

The physicochemical properties were analyzed using ExPASy (https://www.expasy.org/ (accessed on 12 July 2024)) [66]. Subcellular localization prediction of bHLH transcription factor family members was performed using the WoLF PSORT online website (https://wolfpsort.hgc.jp/ (accessed on 12 July 2024)) [67]. We utilized MEME (https://meme-suite.org/meme/) [68] to find the conserved motifs of *DlbHLHs*, with the following parameters: maximum e-value = 1 × 10^−5^; minimum motif length = 6; maximum motif length = 200; and number of motifs = 20. We extracted 2000 bp sequences from each *DlbHLH* gene and used PlantCARE (https://bioinformatics.psb.ugent.be/webtools/plantcare/html/ (accessed on 12 July 2024)) [69] and PlantTFDB (https://planttfdb.gao-lab.org/ (accessed on 12 July 2024)) [70] to predict cis-elements and TFBs. Afterward, we created conserved domain sequence logos and gene structures. The domain analysis result file was obtained by NCBI Batch CD (https://www.ncbi.nlm.nih.gov/Structure/bwrpsb/bwrpsb.cgi/ (accessed on 12 July 2024)). Using the STRING database (http://string-db.org (accessed on 12 July 2024)), we conducted protein interaction analysis and created a protein interaction network based on the *DlbHLH* homologs in *Arabidopsis*.

### 4.6. Phylogeny, Gene Duplications, and Selection Pressure Analysis

To study the evolutionary relationship of *D. latiflorus*, we examined homologs of *DlbHLH* genes in the model plants *Arabidopsis*, *O*. *sativa*, and *T*. *aestivum* using BLAST software v2.10.0 [62] with a maximum e-value of 1 × 10^−20^. These sequences were concatenated with those from *D. latiflorus*, and MUSCLE v5.0 [71] was used to construct the multiple protein sequence alignment, which was then trimmed using trimAL [72]. The IQ-TREE 2 program [73] was used to generate the phylogenetic tree of related proteins. Using the maximum likelihood (ML) method, the bootstrap was set to 1000 and the phylogenetic tree was visualized using iTOL (https://itol.embl.de/) [74].

To assess the degree of overlap between *DlbHLHs* and other species, the annotation file of *the D. latiflorus* genome was used to determine the subgenome assignment, chromosomal location, and exon count of *DlbHLHs* [46]. All *D. latiflorus* proteins were analyzed for similarity using Blast, and data on fragments and tandem repeats were obtained using MSCanX [75]. Moreover, the *DlbHLH* orthologs across species were analyzed in the same way, with the data visualized using NGenomeSyn 1.41 [76]. The genome accession numbers of *D. latiflorus*, *Arabidopsis*, wheat, and rice are JACBGG000000000, GCA_000001735, GCA_900519105, and GCA_001433935. The collinearity among *DlbHLHs* was visualized using the Advanced Circos module in TBtools. The values for non-synonymous substitution (Ka) and synonymous substitution (Ks) in duplicated *DlbHLH* gene pairs were then calculated.

## 5. Conclusions

In this study, 309 members of the *bHLH* gene family were identified in *D. latiflorus*. Based on phylogenetic relationships with the *bHLH* genes in *Arabidopsis*, wheat, and rice, these genes were divided into 23 subfamilies. Structural analysis showed that DlbHLH proteins are relatively conserved in specific subfamilies. Collinearity analysis indicated that the amplification of the *DlbHLH* gene family may be mainly carried out by fragment duplication, some duplicate gene pairs (*DlbHLH21* and *DlbHLH39*) may have functional redundancy, and many duplicate gene pairs (*DlbHLH63* and *DlbHLH124*) show different expression profiles, indicating functional diversification. Many *DlbHLHs* have been identified to be involved in the regulation of flower development. Different homeopathic regulatory elements of hormone response are present in its promoter region, suggesting that *DlbHLH* may be involved in the regulation of flower development. These results laid a foundation for further research on the regulatory mechanism of bHLH protein in the development of bamboo flowers.

## Data Availability

The data presented in this study are available in the article, Supplementary Materials, and online repositories. RNA-seq data used in this work were deposited in the National Genomics Data Center (NGDC) under accession number CRA019186.

## Supplementary Materials

The following supporting information can be downloaded at https://www.mdpi.com/article/10.3390/ijms251910837/s1.

## References

[1] Cho L.-H., Yoon J., An G. (2017). The Control of Flowering Time by Environmental Factors. Plant J..

[2] Tian Z., Liu X., Fan Z., Liu J., Pimm S.L., Liu L., Garcia C., Songer M., Shao X., Skidmore A. (2019). The next Widespread Bamboo Flowering Poses a Massive Risk to the Giant Panda. Biol. Conserv..

[3] Zheng X., Lin S., Fu H., Wan Y., Ding Y. (2020). The Bamboo Flowering Cycle Sheds Light on Flowering Diversity. Front. Plant Sci..

[4] Böhlenius H., Huang T., Charbonnel-Campaa L., Brunner A.M., Jansson S., Strauss S.H., Nilsson O. (2006). *CO/FT* Regulatory Module Controls Timing of Flowering and Seasonal Growth Cessation in Trees. Science.

[5] Keeley J.E., Bond W.J. (1999). Mast Flowering and Semelparity in Bamboos: The Bamboo Fire Cycle Hypothesis. Am. Nat..

[6] Wu C., Cheng Z., Gao J. (2024). Mysterious Bamboo Flowering Phenomenon: A Literature Review and New Perspectives. Sci. Total Environ..

[7] Fornara F., De Montaigu A., Coupland G. (2010). SnapShot: Control of Flowering in *Arabidopsis*. Cell.

[8] Bouché F., Lobet G., Tocquin P., Périlleux C. (2016). FLOR-ID: An Interactive Database of Flowering-Time Gene Networks in *Arabidopsis thaliana*. Nucleic Acids Res..

[9] Ferré-D’Amaré A.R., Prendergast G.C., Ziff E.B., Burley S.K. (1993). Recognition by Max of Its Cognate DNA through a Dimeric b/HLH/Z Domain. Nature.

[10] Hao Y., Zong X., Ren P., Qian Y., Fu A. (2021). Basic Helix-Loop-Helix (bHLH) Transcription Factors Regulate a Wide Range of Functions in Arabidopsis. Int. J. Mol. Sci..

[11] Liu H., Wang K., Yang J., Wang X., Mei Q., Qiu L., Ma F., Mao K. (2023). The Apple Transcription Factor MdbHLH4 Regulates Plant Morphology and Fruit Development by Promoting Cell Enlargement. Plant Physiol. Biochem..

[12] Huang Y., Xing X., Jin J., Tang Y., Ding L., Song A., Chen S., Chen F., Jiang J., Fang W. (2024). CmbHLH110, a Novel bHLH Transcription Factor, Accelerates Flowering in Chrysanthemum. Hortic. Plant J..

[13] Guo H., Yang H., Mockler T.C., Lin C. (1998). Regulation of Flowering Time by *Arabidopsis* Photoreceptors. Science.

[14] Liu H., Yu X., Li K., Klejnot J., Yang H., Lisiero D., Lin C. (2008). Photoexcited CRY2 Interacts with CIB1 to Regulate Transcription and Floral Initiation in *Arabidopsis*. Science.

[15] Liu Y., Li X., Ma D., Chen Z., Wang J., Liu H. (2018). CIB 1 and CO Interact to Mediate CRY 2-dependent Regulation of Flowering. EMBO Rep..

[16] Ledent V., Vervoort M. (2001). The Basic Helix-Loop-Helix Protein Family: Comparative Genomics and Phylogenetic Analysis. Genome Res..

[17] Xiang M., Ding W., Wu C., Wang W., Ye S., Cai C., Hu X., Wang N., Bai W., Tang X. (2021). Production of Purple Ma Bamboo (*Dendrocalamus latiflorus* Munro) with Enhanced Drought and Cold Stress Tolerance by Engineering Anthocyanin Biosynthesis. Planta.

[18] Wang R., Guo Z., Cai C., Zhang J., Bian F., Sun S., Wang Q. (2021). Practices and Roles of Bamboo Industry Development for Alleviating Poverty in China. Clean Technol. Environ. Policy.

[19] Zhu P., Yang J., Yang D., Xu Y., He T., Rong J., Zheng Y., Chen L. (2023). Identification and Characterization of the Cupin_1 Domain-Containing Proteins in Ma Bamboo (*Dendrocalamus latiflorus*) and Their Potential Role in Rhizome Sprouting. Front. Plant Sci..

[20] Taylor A.H., Zisheng Q. (1988). Regeneration from seed of *Sinarundinaria fangiana*, a bamboo, in the wolong giant panda reserve, Sichuan, China. Am. J. Bot..

[21] Yang D., Yang J., Wan J., Xu Y., Li L., Rong J., Chen L., He T., Zheng Y. (2022). Genome-Wide Identification of MIKCc-Type MADS-Box Family Gene and Floral Organ Transcriptome Characterization in Ma Bamboo (*Dendrocalamus latiflorus* Munro). Genes.

[22] Wang X., Wang Y., Yang G., Zhao L., Zhang X., Li D., Guo Z. (2020). Complementary Transcriptome and Proteome Analyses Provide Insight into the Floral Transition in Bamboo (*Dendrocalamus latiflorus* Munro). Int. J. Mol. Sci..

[23] Gao F., Dubos C. (2024). The Arabidopsis bHLH Transcription Factor Family. Trends Plant Sci..

[24] Guo X.-J., Wang J.-R. (2017). Global Identification, Structural Analysis and Expression Characterization of bHLH Transcription Factors in Wheat. BMC Plant Biol..

[25] Yin Y., Yan Z., Guan J., Huo Y., Wang T., Li T., Cui Z., Ma W., Wang X., Chen W. (2023). Two Interacting Basic Helix-Loop-Helix Transcription Factors Control Flowering Time in Rice. Plant Physiol..

[26] Toledo-Ortiz G., Huq E., Quail P.H. (2003). The Arabidopsis Basic/Helix-Loop-Helix Transcription Factor Family[W]. Plant Cell.

[27] Pires N., Dolan L. (2010). Origin and Diversification of Basic-Helix-Loop-Helix Proteins in Plants. Mol. Biol. Evol..

[28] Xu J., Xu H., Zhao H., Liu H., Xu L., Liang Z. (2022). Genome-Wide Investigation of bHLH Genes and Expression Analysis under Salt and Hormonal Treatments in Andrographis Paniculata. Ind. Crops Prod..

[29] Wang R., Li Y., Gao M., Han M., Liu H. (2022). Genome-Wide Identification and Characterization of the bHLH Gene Family and Analysis of Their Potential Relevance to Chlorophyll Metabolism in *Raphanus sativus* L.. BMC Genom..

[30] Fajardo D., Saint Jean R., Lyons P.J. (2023). Acquisition of New Function through Gene Duplication in the Metallocarboxypeptidase Family. Sci. Rep..

[31] Ding A., Ding A., Li P., Wang J., Cheng T., Bao F., Zhang Q. (2021). Genome-Wide Identification and Low-Temperature Expression Analysis of bHLH Genes in Prunus Mume. Front. Genet..

[32] Yu J., Xie Q., Li C., Dong Y., Zhu S., Chen J. (2020). Comprehensive Characterization and Gene Expression Patterns of LBD Gene Family in Gossypium. Planta.

[33] Wittkopp P.J., Kalay G. (2012). Cis-Regulatory Elements: Molecular Mechanisms and Evolutionary Processes Underlying Divergence. Nat. Rev. Genet..

[34] Sasaki-Sekimoto Y., Jikumaru Y., Obayashi T., Saito H., Masuda S., Kamiya Y., Ohta H., Shirasu K. (2013). Basic Helix-Loop-Helix Transcription Factors JASMONATE-ASSOCIATED MYC2-LIKE1 (JAM1), JAM2, and JAM3 Are Negative Regulators of Jasmonate Responses in Arabidopsis. Plant Physiol..

[35] Wang H., Li Y., Pan J., Lou D., Hu Y., Yu D. (2017). The bHLH Transcription Factors MYC2, MYC3, and MYC4 Are Required for Jasmonate-Mediated Inhibition of Flowering in Arabidopsis. Mol. Plant.

[36] Xing S., Quodt V., Chandler J., Höhmann S., Berndtgen R., Huijser P. (2013). SPL8 Acts Together with the Brassinosteroid-Signaling Component BIM1 in Controlling Arabidopsis Thaliana Male Fertility. Plants.

[37] Wu L., Wang K., Chen M., Su W., Liu Z., Guo X., Ma M., Qian S., Deng Y., Wang H. (2024). ALLENE OXIDE SYNTHASE (AOS) Induces Petal Senescence through a Novel JA-Associated Regulatory Pathway in Arabidopsis. Physiol. Mol. Biol. Plants.

[38] Liu Y., Li X., Li K., Liu H., Lin C. (2013). Multiple bHLH Proteins Form Heterodimers to Mediate CRY2-Dependent Regulation of Flowering-Time in *Arabidopsis*. PLoS Genet..

[39] Ito S., Song Y.H., Josephson-Day A.R., Miller R.J., Breton G., Olmstead R.G., Imaizumi T. (2012). FLOWERING BHLH Transcriptional Activators Control Expression of the Photoperiodic Flowering Regulator *CONSTANS* in *Arabidopsis*. Proc. Natl. Acad. Sci. USA.

[40] Kazan K., Manners J.M. (2013). MYC2: The Master in Action. Mol. Plant.

[41] Wang Y., Chen K.-P., Yao Q. (2008). Progress of studies on bHLH transcription factor families. Yi Chuan.

[42] Zhu Z., Liang H., Chen G., Li F., Wang Y., Liao C., Hu Z. (2019). The bHLH Transcription Factor SlPRE2 Regulates Tomato Fruit Development and Modulates Plant Response to Gibberellin. Plant Cell Rep..

[43] Ikeda M., Mitsuda N., Ishizuka T., Satoh M., Ohme-Takagi M. (2021). The CIB1 Transcription Factor Regulates Light- and Heat-Inducible Cell Elongation via a Two-Step HLH/bHLH System. J. Exp. Bot..

[44] Li X., Cao H., Yu D., Xu K., Zhang Y., Shangguan X., Zheng X., Yang Z., Li C., Pan X. (2023). SlbHLH152, a bHLH Transcription Factor Positively Regulates Iron Homeostasis in Tomato. Plant Sci..

[45] Jin K., Wang Y., Zhuo R., Xu J., Lu Z., Fan H., Huang B., Qiao G. (2022). TCP Transcription Factors Involved in Shoot Development of Ma Bamboo (*Dendrocalamus latiflorus* Munro). Front. Plant Sci..

[46] Zheng Y., Yang D., Rong J., Chen L., Zhu Q., He T., Chen L., Ye J., Fan L., Gao Y. (2022). Allele-Aware Chromosome-Scale Assembly of the Allopolyploid Genome of Hexaploid Ma Bamboo (*Dendrocalamus latiflorus* Munro). J. Integr. Plant Biol..

[47] Feller A., Machemer K., Braun E.L., Grotewold E. (2011). Evolutionary and Comparative Analysis of MYB and bHLH Plant Transcription Factors. Plant J..

[48] Cannon S.B., Mitra A., Baumgarten A., Young N.D., May G. (2004). The Roles of Segmental and Tandem Gene Duplication in the Evolution of Large Gene Families in Arabidopsis Thaliana. BMC Plant Biol..

[49] Liang J., Fang Y., An C., Yao Y., Wang X., Zhang W., Liu R., Wang L., Aslam M., Cheng Y. (2023). Genome-Wide Identification and Expression Analysis of the bHLH Gene Family in Passion Fruit (*Passiflora edulis*) and Its Response to Abiotic Stress. Int. J. Biol. Macromol..

[50] Liu M., Qiao G., Jiang J., Yang H., Xie L., Xie J., Zhuo R. (2012). Transcriptome Sequencing and De Novo Analysis for Ma Bamboo (*Dendrocalamus latiflorus* Munro) Using the Illumina Platform. PLoS ONE.

[51] Guo L., Sun X., Li Z., Wang Y., Fei Z., Jiao C., Feng J., Cui D., Feng X., Ding Y. (2019). Morphological Dissection and Cellular and Transcriptome Characterizations of Bamboo Pith Cavity Formation Reveal a Pivotal Role of Genes Related to Programmed Cell Death. Plant Biotechnol. J..

[52] Xu Y., Wong M., Yang J., Ye Z., Jiang P., Zheng S. (2011). Dynamics of Carbon Accumulation During the Fast Growth Period of Bamboo Plant. Bot. Rev..

[53] Goossens J., Mertens J., Goossens A. (2016). Role and Functioning of bHLH Transcription Factors in Jasmonate Signalling. EXBOTJ.

[54] Biswas P., Chakraborty S., Dutta S., Pal A., Das M. (2016). Bamboo Flowering from the Perspective of Comparative Genomics and Transcriptomics. Front. Plant Sci..

[55] Riboni M., Robustelli Test A., Galbiati M., Tonelli C., Conti L. (2016). ABA-Dependent Control of GIGANTEA Signalling Enables Drought Escape via up-Regulation of FLOWERING LOCUS T in Arabidopsis Thaliana. J. Exp. Bot..

[56] Fang Y., Guo D., Wang Y., Wang N., Fang X., Zhang Y., Li X., Chen L., Yu D., Zhang B. (2024). Rice Transcriptional Repressor OsTIE1 Controls Anther Dehiscence and Male Sterility by Regulating JA Biosynthesis. Plant Cell.

[57] Pertea M., Kim D., Pertea G.M., Leek J.T., Salzberg S.L. (2016). Transcript-Level Expression Analysis of RNA-Seq Experiments with HISAT, StringTie and Ballgown. Nat. Protoc..

[58] Liao Y., Smyth G.K., Shi W. (2014). feature Counts: An Efficient General Purpose Program for Assigning Sequence Reads to Genomic Features. Bioinformatics.

[59] Love M.I., Huber W., Anders S. (2014). Moderated Estimation of Fold Change and Dispersion for RNA-Seq Data with DESeq2. Genome Biol..

[60] Chen C., Chen H., Zhang Y., Thomas H.R., Frank M.H., He Y., Xia R. (2020). TBtools: An Integrative Toolkit Developed for Interactive Analyses of Big Biological Data. Mol. Plant.

[61] Liu M., Jiang J., Han X., Qiao G., Zhuo R. (2014). Validation of Reference Genes Aiming Accurate Normalization of qRT-PCR Data in *Dendrocalamus latiflorus* Munro. PLoS ONE.

[62] Camacho C., Coulouris G., Avagyan V., Ma N., Papadopoulos J., Bealer K., Madden T.L. (2009). BLAST+: Architecture and Applications. BMC Bioinform..

[63] Mistry J., Chuguransky S., Williams L., Qureshi M., Salazar G.A., Sonnhammer E.L.L., Tosatto S.C.E., Paladin L., Raj S., Richardson L.J. (2021). Pfam: The Protein Families Database in 2021. Nucleic Acids Res.

[64] Potter S.C., Luciani A., Eddy S.R., Park Y., Lopez R., Finn R.D. (2018). HMMER Web Server: 2018 Update. Nucleic Acids Res..

[65] Paysan-Lafosse T., Blum M., Chuguransky S., Grego T., Pinto B.L., Salazar G.A., Bileschi M.L., Bork P., Bridge A., Colwell L. (2023). InterPro in 2022. Nucleic Acids Res.

[66] Duvaud S., Gabella C., Lisacek F., Stockinger H., Ioannidis V., Durinx C. (2021). Expasy, the Swiss Bioinformatics Resource Portal, as Designed by Its Users. Nucleic Acids Res..

[67] Horton P., Park K.-J., Obayashi T., Fujita N., Harada H., Adams-Collier C.J., Nakai K. (2007). WoLF PSORT: Protein Localization Predictor. Nucleic Acids Res..

[68] Bailey T.L., Johnson J., Grant C.E., Noble W.S. (2015). The MEME Suite. Nucleic Acids Res..

[69] Lescot M. (2002). PlantCARE, a Database of Plant Cis-Acting Regulatory Elements and a Portal to Tools for in Silico Analysis of Promoter Sequences. Nucleic Acids Res..

[70] Guo A.-Y., Chen X., Gao G., Zhang H., Zhu Q.-H., Liu X.-C., Zhong Y.-F., Gu X., He K., Luo J. (2007). PlantTFDB: A Comprehensive Plant Transcription Factor Database. Nucleic Acids Res..

[71] Edgar R.C. (2004). MUSCLE: Multiple Sequence Alignment with High Accuracy and High Throughput. Nucleic Acids Res..

[72] Capella-Gutiérrez S., Silla-Martínez J.M., Gabaldón T. (2009). trimAl: A Tool for Automated Alignment Trimming in Large-Scale Phylogenetic Analyses. Bioinformatics.

[73] Minh B.Q., Schmidt H.A., Chernomor O., Schrempf D., Woodhams M.D., Von Haeseler A., Lanfear R. (2020). IQ-TREE 2: New Models and Efficient Methods for Phylogenetic Inference in the Genomic Era. Mol. Biol. Evol..

[74] Letunic I., Bork P. (2021). Interactive Tree of Life (iTOL) v5: An Online Tool for Phylogenetic Tree Display and Annotation. Nucleic Acids Res..

[75] Wang Y., Tang H., Wang X., Sun Y., Joseph P.V., Paterson A.H. (2024). Detection of Colinear Blocks and Synteny and Evolutionary Analyses Based on Utilization of MCScanX. Nat. Protoc..

[76] He W., Yang J., Jing Y., Xu L., Yu K., Fang X. (2023). NGenomeSyn: An Easy-to-Use and Flexible Tool for Publication-Ready Visualization of Syntenic Relationships across Multiple Genomes. Bioinformatics.

